# OXSA: An open-source magnetic resonance spectroscopy analysis toolbox in MATLAB

**DOI:** 10.1371/journal.pone.0185356

**Published:** 2017-09-22

**Authors:** Lucian A. B. Purvis, William T. Clarke, Luca Biasiolli, Ladislav Valkovič, Matthew D. Robson, Christopher T. Rodgers

**Affiliations:** 1 Oxford Centre for Clinical Magnetic Resonance Research (OCMR), University of Oxford, John Radcliffe Hospital, Oxford, United Kingdom; 2 Department of Imaging Methods, Institute of Measurement Science, Slovak Academy of Sciences, Bratislava, Slovakia; National Research Council of Italy, ITALY

## Abstract

In vivo magnetic resonance spectroscopy provides insight into metabolism in the human body. New acquisition protocols are often proposed to improve the quality or efficiency of data collection. Processing pipelines must also be developed to use these data optimally. Current fitting software is either targeted at general spectroscopy fitting, or for specific protocols. We therefore introduce the MATLAB-based OXford Spectroscopy Analysis (OXSA) toolbox to allow researchers to rapidly develop their own customised processing pipelines. The toolbox aims to simplify development by: being easy to install and use; seamlessly importing Siemens Digital Imaging and Communications in Medicine (DICOM) standard data; allowing visualisation of spectroscopy data; offering a robust fitting routine; flexibly specifying prior knowledge when fitting; and allowing batch processing of spectra. This article demonstrates how each of these criteria have been fulfilled, and gives technical details about the implementation in MATLAB. The code is freely available to download from https://github.com/oxsatoolbox/oxsa.

## Introduction

Magnetic resonance spectroscopy (MRS) aims to quantify metabolites to understand the biochemistry of the body [1]. Many different MRS acquisition methods have been proposed. Some are optimized for speed [2], and others for better signal localization or signal-to-noise ratio (SNR) (e.g. Table 1 in ref. [3]). Furthermore, methods have been developed to measure more than just metabolite concentrations (e.g. saturation transfer methods to measure chemical exchange rates [4, 5]).

All of these data need to be processed (i.e. generate results), and to this end many different fitting software solutions have been developed, e.g., LCModel [6], jMRUI [7], TARQUIN [8], ProFit [9]. However, most fitting packages either aim to be a one-size-fits-all solution or to answer one specific problem, which necessarily limits the freedom of researchers investigating new MRS techniques. In order to get the most robust, accurate and useful results, the data processing strategy should be optimised for the chosen acquisition method.

We therefore introduce the OXford Spectroscopy Analysis toolbox (OXSA) to simplify the tailored development of fully automated or semi-automated processing pipelines by:

1. Being easy to install and use
2. Seamlessly importing Siemens Digital Imaging and Communications in Medicine (DICOM) standard data, including all of the measurement headers
3. Allowing visualisation of 1, 2 and 3D-resolved spectroscopy data volumes of interest (VOIs) overlaid on anatomical localizer images
4. Offering a robust time-domain analysis (i.e. fitting) routine (an implementation of the *advanced method for accurate*, *robust and efficient spectral fitting* (AMARES) algorithm)
5. Flexibly and dynamically specifying prior knowledge when fitting
6. Allowing batch processing of spectra

This tool was originally developed in the context of our phosphorus (^31^P) MRS studies in the human heart and liver, processing data acquired on Siemens MR systems. It has subsequently been used to process proton (^1^H), carbon (^13^C) and ^31^P spectra in a range of organs.

## Tool installation and dependencies

The OXSA code is available for download (https://github.com/oxsatoolbox/oxsa) under an academic license. This also includes an example of a compiled tool built using OXSA, for distribution to computers without a MATLAB license. The example is adapted for ^31^P-MRS analysis. Example code is provided to create other compiled versions. The OXSA code has been used for analysis of ^31^P chemical shift imaging (CSI) data at the University of Oxford, the University of Edinburgh, the University of Minnesota, Auburn University, Raboud University, McMaster University and the Medical University of Vienna. OXSA-based processing pipelines are routinely used for processing of ^1^H and ^13^C MRS data in Oxford.

The tool was developed in MATLAB, which allows for straightforward setup and immediate use. The tool can be run immediately after downloading the code, with no further installation required. In addition, the graphical user interface (GUI) and debugging tools allow simple development of new algorithms and processing pipelines, as well as combination with already existing, in-house processing tools. The code runs on Windows, Linux and Mac OS, and has been tested for MATLAB R2014a to R2017a.

The open-source dcm4che java toolkit is used to load in DICOM files [10]. A working version of this toolkit is included with the OXSA code, thus no further downloads are necessary. The only additional requirements are the MATLAB Optimization and Image Processing toolboxes. The Symbolic Math Toolbox is also required if AMARES.estimateDerivedParamAndCRB is used for a new functional form, but generated m-code for the analytical Jacobian is saved for all future invocations. No MEX files are required.

Requests for bug-fixes for the OXSA code can be reported using GutHub’s issue framework, which can be found at https://github.com/OXSAtoolbox/OXSA/issues.

## Importing data

**Fig 1:**
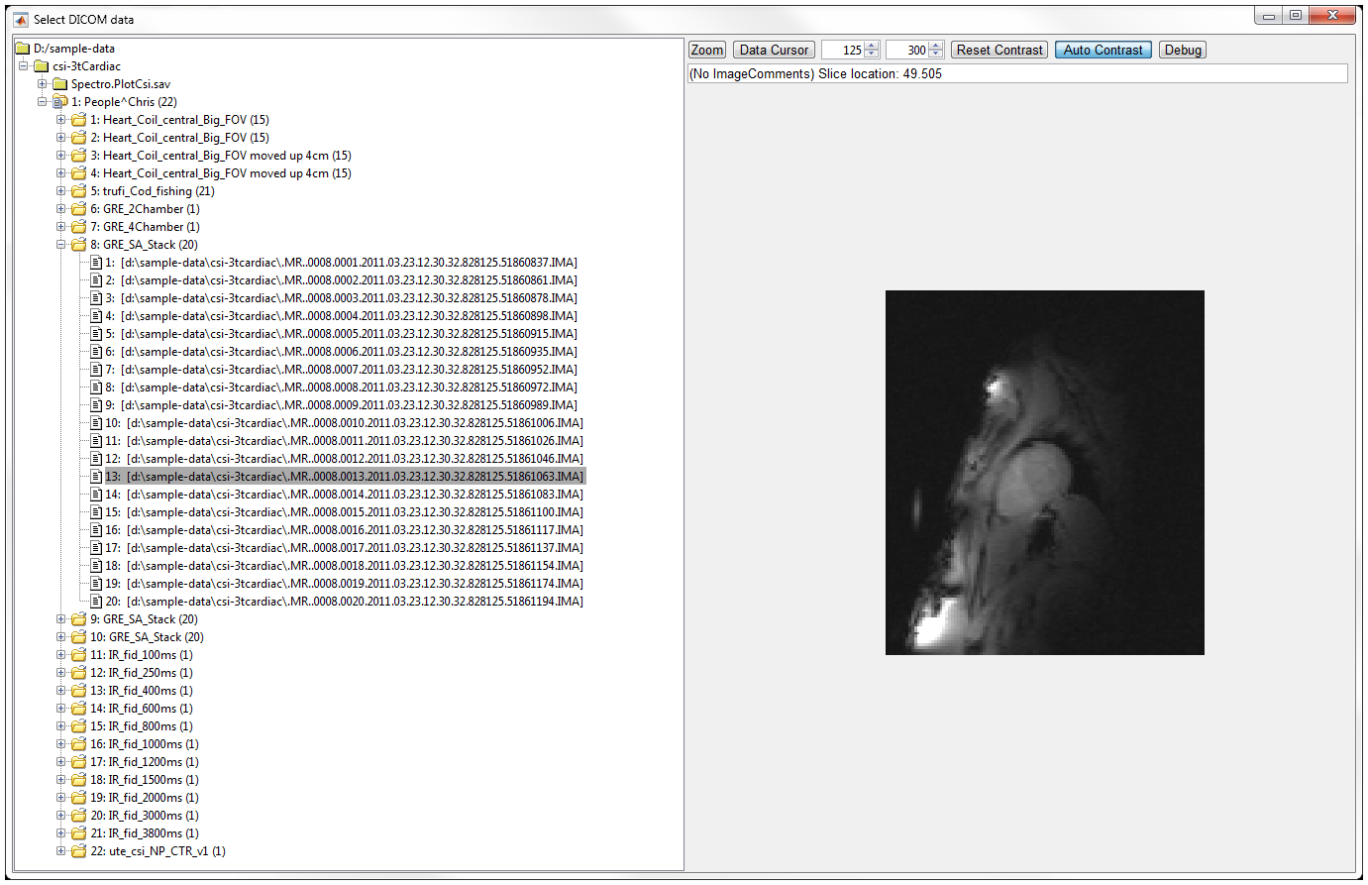
The OXSA Siemens DICOM loading GUI. A Spectro.FileOpenGui object showing a Spectro.dicomUiTree (left) together with a DICOM image preview (right). The contrast can be adjusted in the top right, and individual pixels can be examined using the data cursor.

The classes used for loading DICOM data are given in Table 1. The dicomTree class checks for DICOM or DICOMDIR files in the chosen folder or nested folders, on local or network drives, and sorts them into a nested structure. It then saves a cache in a.mat file in the local system’s temporary directory to allow very rapid scanning of folders for new files on subsequent occasions. The dicomTree class has helper methods that are used to search the tree for target DICOM data. Alternatively, an interactive GUI DICOM selector is provided (see Fig 1). Once found, this data can be loaded into MATLAB using the Spectro.dicom class or subclasses Spectro.Spec and Spectro.dicomImage that are specialised for spectroscopy and imaging data respectively. These classes call the SiemensCsaParse function to parse the Siemens DICOM headers, including the private Common Syngo Architecture (CSA) headers, and to organise the data into a MATLAB structure.

**Table 1:** OXSA data and GUI classes.

Class name	Use
Spectro.dicomTree	Scans directory for DICOM or DICOMDIR files
Spectro.dicomUiTree	GUI element for selection of DICOM study/series/instance.
Spectro.FileOpenGui	Dialog window showing a Spectro.dicomUiTree together with image preview.
Spectro.dicom	Root of the hierarchy. Methods to decode Siemens private DICOM headers.
- Spectro.dicomImage	Methods for interpretation and plotting of DICOM images.
- Spectro.Spec	Base class for handling of spectroscopy DICOM files.
Spectro.PlotCsi	Base class for spectroscopy GUI.

## Visualising spectroscopy data

**Fig 2:**
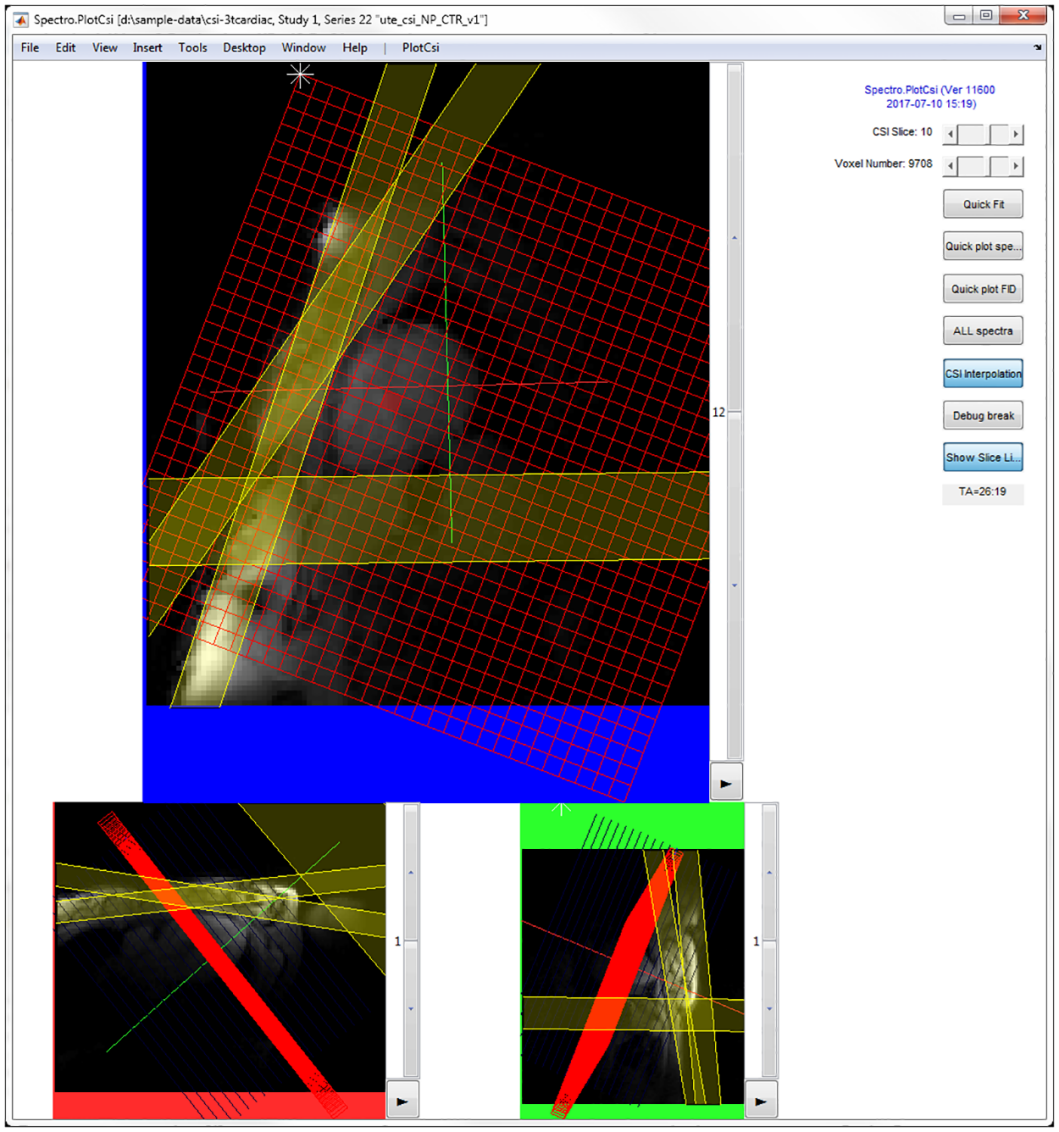
Spectroscopy visualisation tool. The red lines mark the nominal voxel size. The selected voxel is highlighted in red. Saturation bands are highlighted in yellow. The sliders on the right hand side can be used to select CSI slice or voxel. The buttons can be used to run various functions to e.g. fit or inspect the data from a single voxel.

Spectroscopy data can be visualised using the Spectro.PlotCsi GUI (see Fig 2). The GUI works for 1, 2 and 3D CSI, as well as single-voxel or non-localized data. The individual subplots show by default the image stacks that were in the viewer when the spectra were acquired. A red grid is overlaid that shows the location of the voxel or voxels acquired with the sequence, and the selected voxel is highlighted in red. Saturation bands can also be shown, highlighted in yellow. The perpendicular image stacks are shown in blue and green, with the current images highlighted. The contrast of each of the images can be adjusted, and a new stack of localizer images can be loaded interactively. There are buttons for calling the real and imaginary spectra and free-induction decays (FIDs) from the selected voxel, for turning CSI interpolation on and off, and for calling the fitting algorithm. The GUI tool currently assumes reconstructed Cartesian layout of voxels in real space.

## Fitting algorithm

The simplest method of quantitation involves peak area integration in the frequency-domain spectrum. However, underlying broad resonances, overlapping and low signal-to-noise ratio peaks, and operator error can cause biased or inaccurate results [11]. The fitting algorithm we have used is based on the *advanced method for accurate*, *robust and efficient spectral fitting* (AMARES), which is a popular time-domain fitting algorithm that incorporates flexible prior knowledge to improve the accuracy and precision of fitting in-vivo magnetic resonance spectra [12]. AMARES has been used for fitting phosphorus (^31^P) [13], proton (^1^H) [14] and carbon (^13^C) [15] MRS data.

The AMARES algorithm fits data in a linear least-squares sense to the following model function [12]:

$$y_{n}={\hat{y}}_{n}+e_{n}=\sum_{k=1}^{k}a_{k}e^{i\varphi_{k}}e^{\left(-d_{k}(1-g_{k}+g_{k}t_{n})t_{n}\right)}e^{i2\pi f_{k}t_{n}}+e_{n}$$ (1)

Where n = 0 to N-1, N is the number of measured points, y_n_ is a sum of exponentially damped sinusoids, i = √-1, a_k_ is the amplitude, φ_k_ is the phase, d_k_ is the damping factor, and f_k_ is the frequency of the k^th^ sinusoid. K is the total number of sinusoids, including one for each component of multiplet peaks. t_n_ = nΔt + t_0_ with Δt sampling interval, and t_0_ time before the first data point. e_n_ is complex white Gaussian noise. The caret on the y indicates that this quantity represents the model function rather than actual measurements. g_k_ denotes whether the lineshape for each peak is Gaussian (g_k_ = 1) or Lorentzian (g_k_ = 0).

The model function variables are chemical shift, linewidth, amplitude (i.e. area) and phase for each peak. They are constrained by prior knowledge comprising the initial values, upper and lower bounds and intrinsic relationships between the peaks. For example, with broadband excitation and after 1^st^-order phase correction to compensate for the receiver dead-time, the spectral peaks often all have the same phase. Multiplet peaks are another example of an intrinsic relationship, as they have a fixed difference in chemical shift based on literature J-couplings and fixed amplitude ratios.

The first step of the fitting process is to solve the least-squares problem for amplitudes and phases using the starting values provided for the frequencies and dampings. The resulting amplitudes and phases are then used as the starting values for the main fitting step. The prior knowledge is applied using a linearized constraint matrix. The fit results are given with estimated Cramér-Rao lower bounds (CRLB), i.e. a measure of the uncertainty in the fit [16].

**Fig 3:**
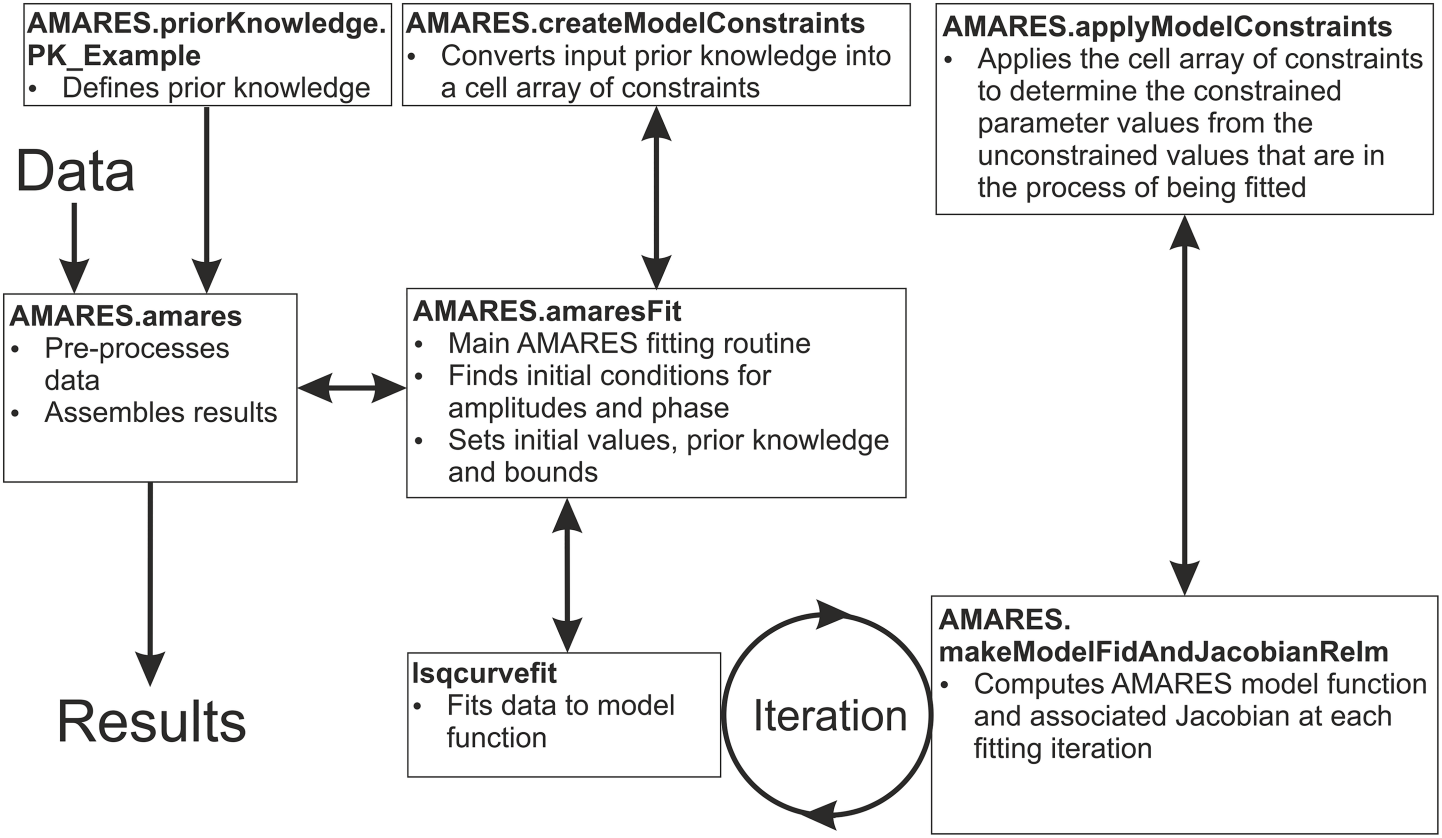
OXSA fitting flow diagram. Flow diagram of the functions used for fitting spectroscopy data that has been loaded into MATLAB.

**Fig 4:**
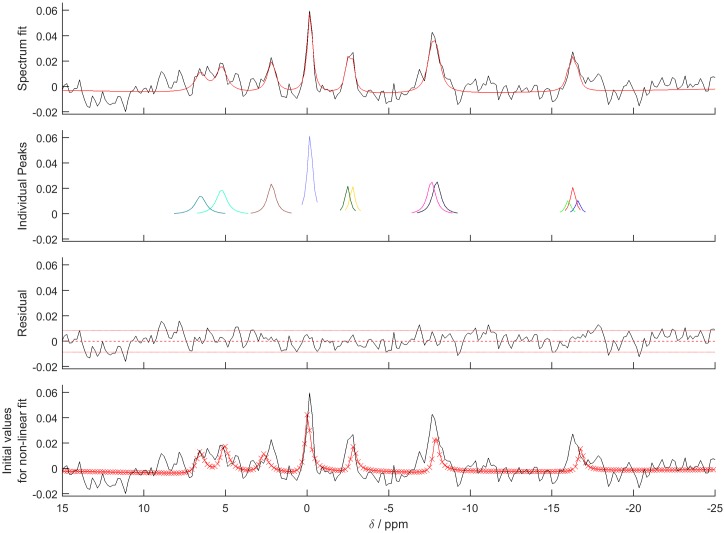
amaresPlot output. The default output figure from amaresPlot showing the fit of the example cardiac ^31^P data. Individual peaks are truncated in the frequency domain to 2.5× the full width at half maximum for display purposes only, i.e. to allow easier identification of peaks in cases where one peak has a much smaller amplitude than the others.

The functions involved in the fitting process are described in Fig 3, and the fitting itself is done by the Trust-Region-Reflective algorithm [17] from MATLAB's lsqcurvefit function. See Fig 4 for an example output. The fitting algorithm was validated against the Java-based magnetic resonance user interface (jMRUI) implementation of AMARES [18]. A detailed description of the validation is given in the S1 Appendix. Forty-five in vivo ^31^P-MRS cardiac datasets were fitted with our OXSA MATLAB AMARES and with jMRUI AMARES. The datasets were acquired under a standard operating procedure approved by UK National Research Services, and in accordance with the Declaration of Helsinki. Excellent correlation (R > 0.99) was observed between the peak parameters of PCr and γ-ATP fitted by jMRUI and OXSA. Low SNR PDE showed lower correlation, though always above 0.7. The average time to run a single voxel from an in vivo cardiac CSI dataset was 18.5 ± 0.3s for jMRUI and 0.69 ± 0.1s for OXSA.

The fitting CRLBs are calculated with the covariance between the parameters. This allows more accurate CRLBs to be estimated for any combinations of the fit results using AMARES.estimateDerivedParamAndCRB. The first time this code is run, the Symbolic Toolbox is used to process the input. For example, '(PCR_am / PCR_sat) / ((ATP_GAMMA1_am + ATP_GAMMA2_am) /ATP_GAMMA_sat)' is processed to give the saturation-corrected PCr/γ-ATP ratio. The generated MATLAB m-code functions that are created are then saved in a.mat cache file. Every subsequent call to AMARES.estimateDerivedParamAndCRB uses this cache to identify the function that correspond to a given string, and the Symbolic Toolbox is therefore no longer required (except on the first run with a new set of prior knowledge e.g. after a new metabolite has been added). This allows for a significant increase in processing speed, and makes it possible to use the MATLAB Compiler to deploy the processing tools as a.EXE executable file for use on computers without MATLAB. An example of calling this function is given from Line 73 of examples/example_fitSimSpec.

## Inclusion of additional prior knowledge

Each basic prior knowledge file, e.g. AMARES.priorKnowledge.PK_7T_Cardiac, gives upper and lower bounds on each fitting parameter, starting values, and the relationship between each peak. In the initial prior knowledge, the possible peak relationships include whether there are multiplet splitting and amplitude ratios, the grouping of linewidths or amplitudes, and the offsets of phase and chemical shift. In AMARES.priorKnowledge.PK_7T_Cardiac_t2, an additional parameter (‘base_linewidth’) is included which gives information about the relative linewidths between peaks. This prior knowledge is then processed by AMARES.createModelConstraints (see Fig 3) to give function handles relating each parameter. In this case, the function required to include the additional linewidth constraint simply adds the base_linewidth parameter to the linewidth parameter used for fitting.

## Example of batch processing of data

As the loading and fitting functions can all be called in MATLAB without the need for a GUI, batch processing of data simply requires writing a short script for iterating through filenames, series or voxels as desired. An example of this is included in the package—examples/example_loadCsiData_batchProcessVoxels. In this script, a CSI phantom dataset is loaded, each voxel is fitted, and an amplitude map is plotted. The batch processing requires a single extra line of code compared to fitting a single voxel.

## Example of a complete processing pipeline

Our group has been using this toolbox to create several complete processing pipelines. For example, pipelines have been created to correct the fitted peaks for partial saturation, blood contamination, and calculate peaks ratios [19, 20]; to calculate B_1_^+^ maps using a Bloch-Siegert method [21]; to automatically perform quality assurance on CSI voxels before and after fitting [22]; and to calculate the creatine kinase exchange rates in the heart [23].

## Discussion

We present an intuitive, easy-to-use open source MRS processing tool: OXSA. It has all the features necessary to process Siemens DICOM spectroscopy data. It is flexible, and can be applied to novel acquisition protocols. In addition, it can be compiled for non-technical users, who do not have access to MATLAB.

The tool is easy to install and use. The user must only have MATLAB, the Image Processing and Optimization toolboxes, and then the code can be run immediately. We have chosen a MATLAB implementation because of MATLAB’s wide availability in academic centres.

Currently, the tool is fully integrated only for Siemens DICOM data. However, there have been open-source MATLAB scripts developed to work for data from other major MR vendors, e.g. Philips, GE. Our flexible code would allow simple integration of these scripts. Similarly, offline reconstruction algorithms can be written in MATLAB and integrated in the OXSA toolbox. This allows simpler development of novel k-space acquisition strategies for CSI, as the reconstruction can be tested in MATLAB before it is added to online scanner reconstruction.

At present, our toolbox allows the visualisation of a 1D spectrum that is localized in 1, 2, or 3 dimensional space. If spectra were acquired with an additional spectral or temporal dimension, different methods of visualisation could be added to the toolbox.

In this implementation, a fitting algorithm based on AMARES has been used. The results of the validation showed that it correlates well with the implementation of AMARES in jMRUI, with a 96.3% reduction in fitting time. This is due to multi-threaded acceleration of the critical non-linear least squares fitting steps in MATLAB.

As with all fitting algorithms, a more complicated spectrum is more challenging to fit. Whereas methods like LCModel use metabolite basis sets to simplify this problem [6], the OXSA package, like the AMARES algorithm it is based on, relies on increasing the amount of prior knowledge to achieve a good fit. In cases where there are many overlapping peaks and a complex baseline, e.g., short TE brain MR spectra, the prior knowledge can be time-consuming to set up correctly. However, once this is achieved even very complex spectra can be fitted correctly [14].

The fitting algorithm could be adapted e.g. by changing the lineshape or the fitting domain. This would allow the fitting to match the acquisition parameters optimally. The inclusion of additional prior knowledge has been demonstrated with a relative linewidth constraint. Extending this further, it would be possible to constrain the linewidths or chemical shift across multiple datasets. This would be useful in spectroscopy with additional dimensions, such as a stress-response or saturation-recovery studies. It would also be possible to smooth linewidth variation in neighbouring voxels, based on the assumption of a spatially smooth B_0_ inhomogeneity [24].

Batch processing is an important step towards a completely automated, and operator-independent processing pipeline. The example included in the package, examples/example_loadCsiData_batchProcessVoxels, processes multiple voxels from a single CSI dataset, but with minimal additions, this code could be extended to process multiple series within a scan, or multiple different subjects.

Clinical MRS is currently limited by complicated processing, and hence a requirement for expert users. Fully-automated, operator-independent processing pipelines that compute processed results in one step from raw data simplify the MRS processing and minimize the chance for user error. This toolbox will make it straightforward for researchers to produce their own, personalized, processing pipelines.

## Conclusion

We have developed a simple-to-use toolbox to visualise and process Siemens spectroscopy data in MATLAB. The toolbox allows the batch processing of single voxel spectra as well as 1D, 2D and 3D CSI datasets. It has an integrated fitting pipeline that can include additional prior knowledge. This toolkit will give researchers more freedom to rapidly develop customised fitting and processing pipelines best suited to their acquisition protocols.

## Supporting information

S1 Appendix Fitting algorithm validation. (DOCX)
