# Supplementary material for: OXSA: An open-source magnetic resonance spectroscopy analysis toolbox in MATLAB
Source: PLoS One. 2017 Sep 22;12(9):e0185356. doi: 10.1371/journal.pone.0185356 (PMC5609763; doi:10.1371/journal.pone.0185356)
Supplement: S1 Appendix — (DOCX) [file pone.0185356.s001.docx]

# S1 Appendix: Fitting algorithm validation

## Methods

To enable automatic comparison of the OXSA fitting algorithm and jMRUI AMARES, a Java (Oracle, Redwood City, CA, USA) test framework, operable from MATLAB, was created to simulate user input to jMRUI. The process may be summarised as follows:

1. Data is loaded in from DICOM files by our MATLAB code.
2. Selected data, one or more voxels, is exported to text file in jMRUI-readable format.
3. A new instance of jMRUI is started by the MATLAB/Java test framework and the data from the text file loaded by jMRUI.
4. Any phasing, apodization and frequency offsets may be applied to the data.
5. The AMARES quantitation module is opened, prior knowledge files loaded and the quantitation run. The prior knowledge may be updated dynamically if required (e.g. for different begin times) by MATLAB code that alters the jMRUI prior-knowledge (.pk, .sv, or .op) binary files.
6. The jMRUI AMARES results are saved in text format.
7. These results are read in MATLAB.

### Single peak

500 synthetic time-domain signals, the Fourier pairs to a single on-resonance singlet peak with an amplitude of 10, a linewidth of 10 Hz and 0° phase, were generated in a Monte-Carlo fashion for eight levels of Gaussian noise with variance stepped from 10^-4^ to 10^3^ (see Supporting Fig S1). Each of the 4000 simulated spectra were fitted in both programs, and the total fitting time, including sending and loading the data into the driving MATLAB script, was recorded using MATLAB’s inbuilt stopwatch function.


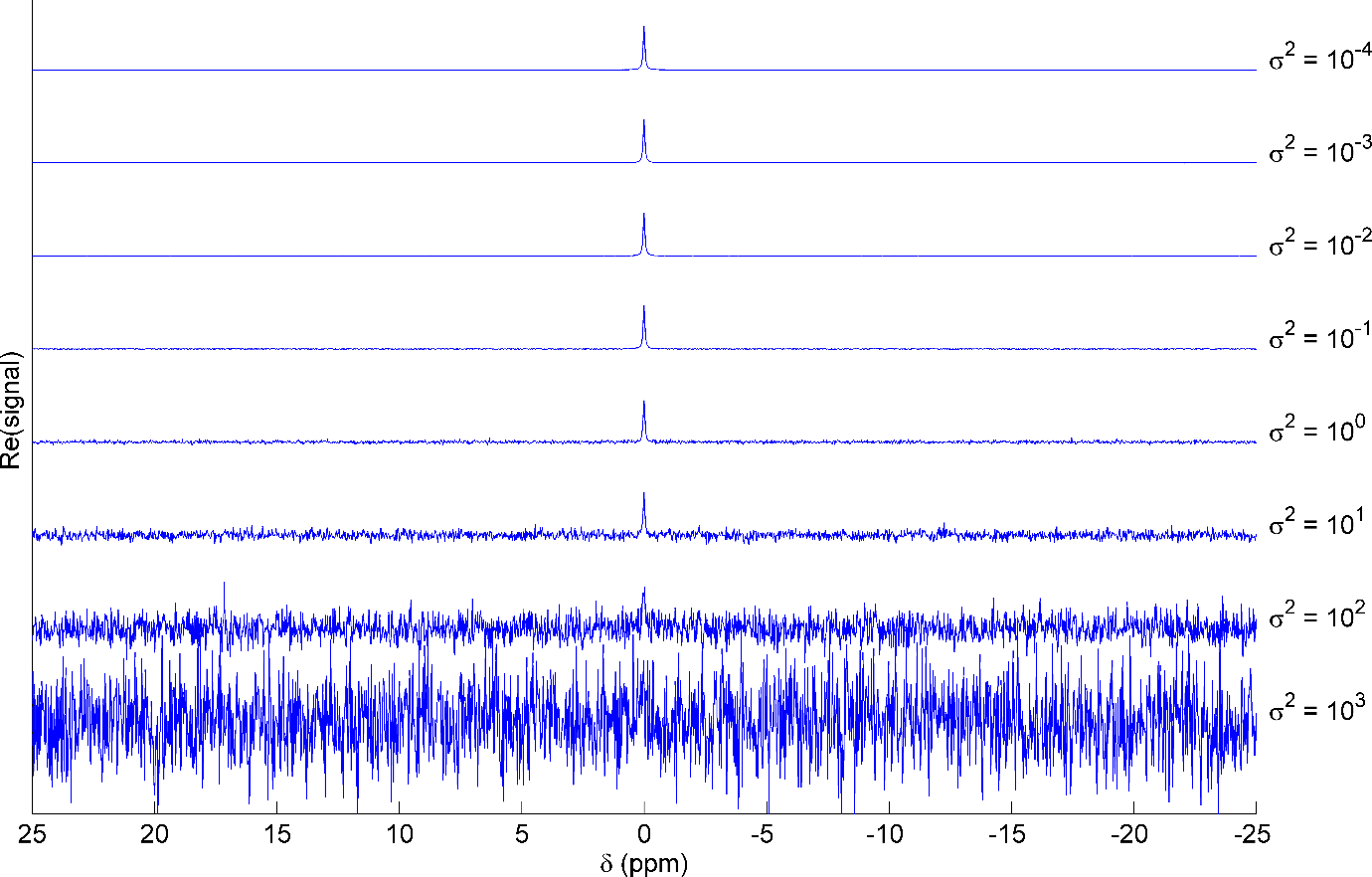


**S1 Fig. Synthetic singlet spectra with added noise.** Example frequency-domain synthetic data of a singlet on-resonance peak. Each spectrum is a single example of the 500 spectra generated per noise level. The variance of the real and complex parts of the noise is denoted on the right of the figure.

### Multiplet peaks

The Monte Carlo test was repeated for on resonance, symmetrical doublet and triplet peaks. The coupling constant, J, was 14Hz in each case.

### Cardiac spectra

A model cardiac spectrum was simulated using the following parameters: equal phases for all peaks, literature chemical shift values and linewidths (1), and a [PCr] / [ATP] ratio of 2, which is typical for a healthy human heart. Gaussian noise was added to the perfect spectrum to give an SNR of 14. The fitting time for 3000 simulated cardiac spectra was recorded for both Matlab and jMRUI driven automatically.

Spectra from the mid-interventricular septal voxel of 45 cardiac ^31^P-MRS datasets were acquired at 3T and 7T under a standard operating procedure approved by the UK National Research Services, and in accordance with the Declaration of Helsinki, as described previously(2). They were fitted using both methods. The ^31^P cardiac prior knowledge was identical for both methods (see Supporting Table S1). The linewidths were unconstrained. The fitted parameters and Cramér-Rao lower bounds (CRLBs) (3) from jMRUI and OXSA were compared across three peaks in the spectrum (PCr, γ-ATP, PDE), representing high, medium and low relative SNR. The total time taken to run each dataset, including loading into jMRUI and fitting, was recorded.

|  | **β-ATP** | **α-ATP** | **γ-ATP** | **PCr** | **PDE** | **DPG-1** | **DPG-2** |
| --- | --- | --- | --- | --- | --- | --- | --- |
| **Multiplet** | | | | | | | |
| Ratio | 1:2:1 | 1:1 | 1:1 | - | - | - | - |
| Splitting (Hz) | 15 | 16 | 15 | - | - | - | - |
| **Initial Values** **(Bounds)** | | | | | | | |
| Chemical shift (ppm) | -16.74 (-inf, inf) | -7.88 (-inf, inf) | -2.82  (-inf, inf) | 0  (-inf, inf) | 2.69  (-inf, inf) | 5.1  (3.5,7.5) | 6.58  (4.0, 8.0) |
| Linewidth (Hz) | 10 (0, inf) | 10 (0, inf) | 10 (0, inf) | 10 (0, inf) | 20 (20,100) | 20 (5,100) | 20 (5,100) |
| Amplitude | 1 (0, inf) | 1 (0, inf) | 1 (0, inf) | 1 (0, inf) | 1 (0, inf) | 1 (0, inf) | 1 (0, inf) |
| Phase (degrees) | 0 (0, 360) | 0 (0, 360) | 0 (0, 360) | 0 (0, 360) | 0 (0, 360) | 0 (0, 360) | 0 (0, 360) |
| **S1 Table. Prior knowledge used in fitting validation.** List of prior knowledge used for OXSA and jMRUI AMARES. The phases of all peaks were additionally constrained to be the same as all the other peaks. | | | | | | | |

## Results

### Single peak

Both OXSA and jMRUI correctly estimated the parameters of the single peak for noise variances less than 10^3^. At the 10^3^ level both methods demonstrated (<20%) bias in fitting the amplitude (i.e. area) and linewidth parameters (see Supporting Fig S2).

##
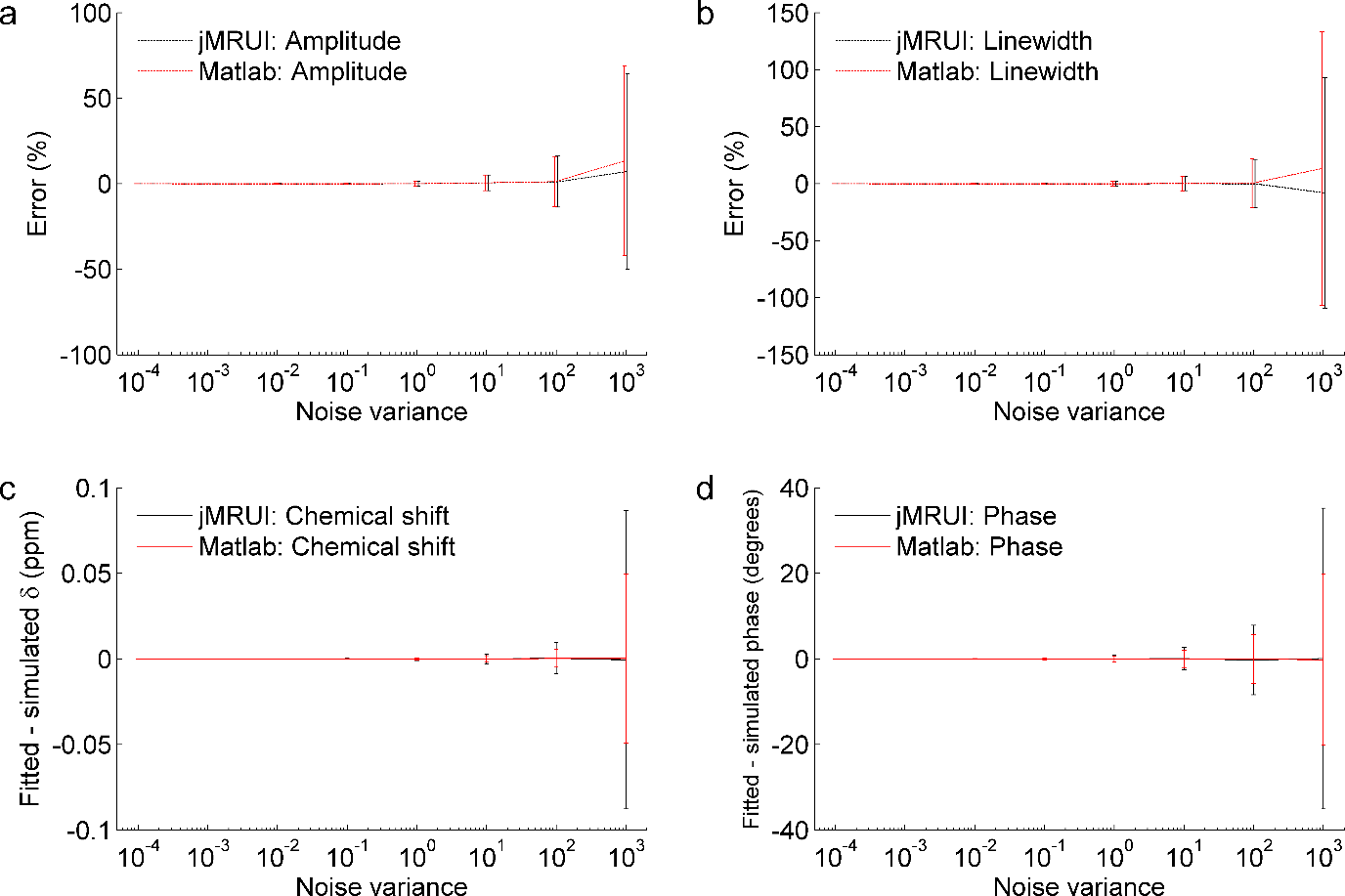


**S2 Fig. Comparison of singlet peak fitting for jMRUI and OXSA.** Monte Carlo comparison of the fitted parameters (a: amplitude, b: linewidths, c: chemical shifts and d: phase) of a single on-resonance singlet peak at varying noise levels for jMRUI and Matlab (OXSA). The values are plotted as the mean error (amplitude and linewidth) or difference (chemical shift and phase) between the fitted and simulation input values of 500 independently fitted spectra per noise level. The standard deviation of the values are plotted as error bars.

Below the 10^3^ noise level, the mean CRLB of each fitted parameter estimated by the OXSA program matched (within 1%) the standard deviation of the fitted parameters of both jMRUI and OXSA and the values predicted by Cavassila et al (3) for isolated singlets. At the 10^3^ noise level, the standard deviation of the jMRUI and OXSA fits deviated (by < 10%) from the CRLB estimates and the literature values.

The mean CRLB estimated by jMRUI were initially not found to match the standard deviation of the fitted parameters as estimated by jMRUI or OXSA, or the predicted values of Cavassila et al. However, dividing the CRLB by the estimated noise, corrects the jMRUI CRLBs to the expected values (see Supporting Fig S3). This was later confirmed as a bug in private communication with the developers of jMRUI. The bug is not present in version 5 of jMRUI, but is thought to be present in versions prior to V4.


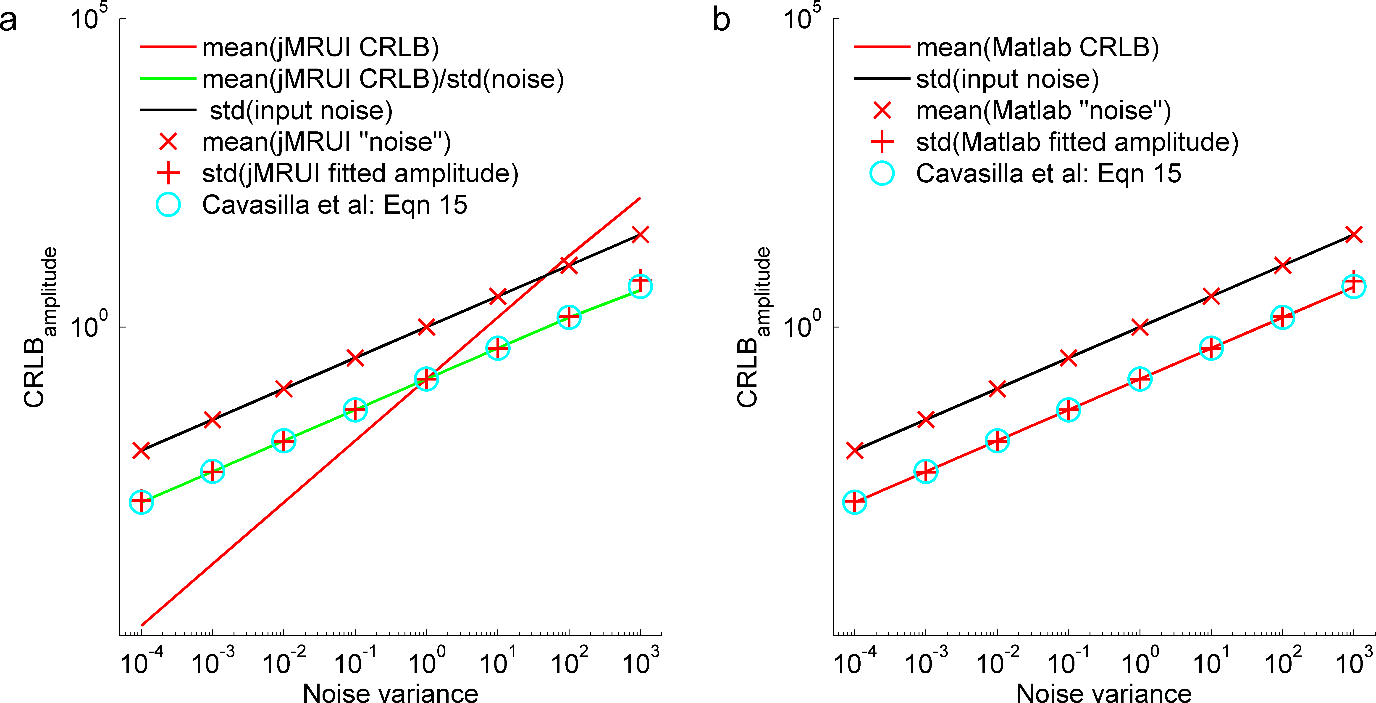


**S3 Fig. Comparison of CRLB from jMRUI and OXSA.** Comparison of the amplitude CRLB of a single isolated peak, estimated by jMRUI (a) and Matlab (OXSA) (b), with the standard deviation of the fitted parameters and the analytical expression given by Equation 15 in reference (4). The CRLB estimated by jMRUI was divided by the noise estimate to correct a confirmed bug. Additionally the standard deviation of the baseline noise estimated by both implementations is compared with the standard deviation of noise generated in the simulation.

The total fitting time for the 4000 single peak spectra was 91s for the automatic jMRUI program and 80s for OXSA.

### Multiplet peaks

Both jMRUI and OXSA showed no error in fitting peak parameters for doublets or triplets at the fixed separation of 14 Hz at noise levels below 10^2^. At 10^2^ a low (< 1%) mean systematic error in amplitude and linewidth was observed in both jMRUI and OXSA, at higher noise levels larger errors were observed with both implementations for all estimated parameters.

The CRLB estimates calculated by OXSA were within 1% of the standard deviation of the fitted parameters, and the values predicted by Cavassila et al, at all noise levels. At noise levels above 10^1^ some divergence from the standard deviation of the fitted parameters was observed, though this was low (<10%) until 10^3^ where the values of the CRLB and the standard deviation diverged by up to 50%.

Total fitting times of the 4000 multiplet signals were 103 s and 95 s for jMRUI and OXSA respectively.

### Cardiac spectra

The fitting time for 3000 simulated cardiac spectra was 1340s for automatic jMRUI compared with 85s for OXSA.

In vivo, excellent correlation (R > 0.99) was observed between the peak parameters of PCr and γ-ATP fitted by jMRUI and OXSA (Supporting Fig S4). Low SNR PDE showed lower correlation, though always above 0.7. Low (<10%), but statistically significant bias between the methods was found for amplitude and linewidths for all peaks (t-test, α = 0.05). No significant bias was seen for chemical shift and phase (α = 0.05). SNR was 23.0 ± 12.9 for PCr, 12.7 ± 6.6 for γ-ATP and 4.1 ± 1.5 for PDE. The average time to run a single voxel from an in vivo cardiac CSI dataset was 18.5 ± 0.3s for jMRUI and 0.69 ± 0.1s for OXSA.


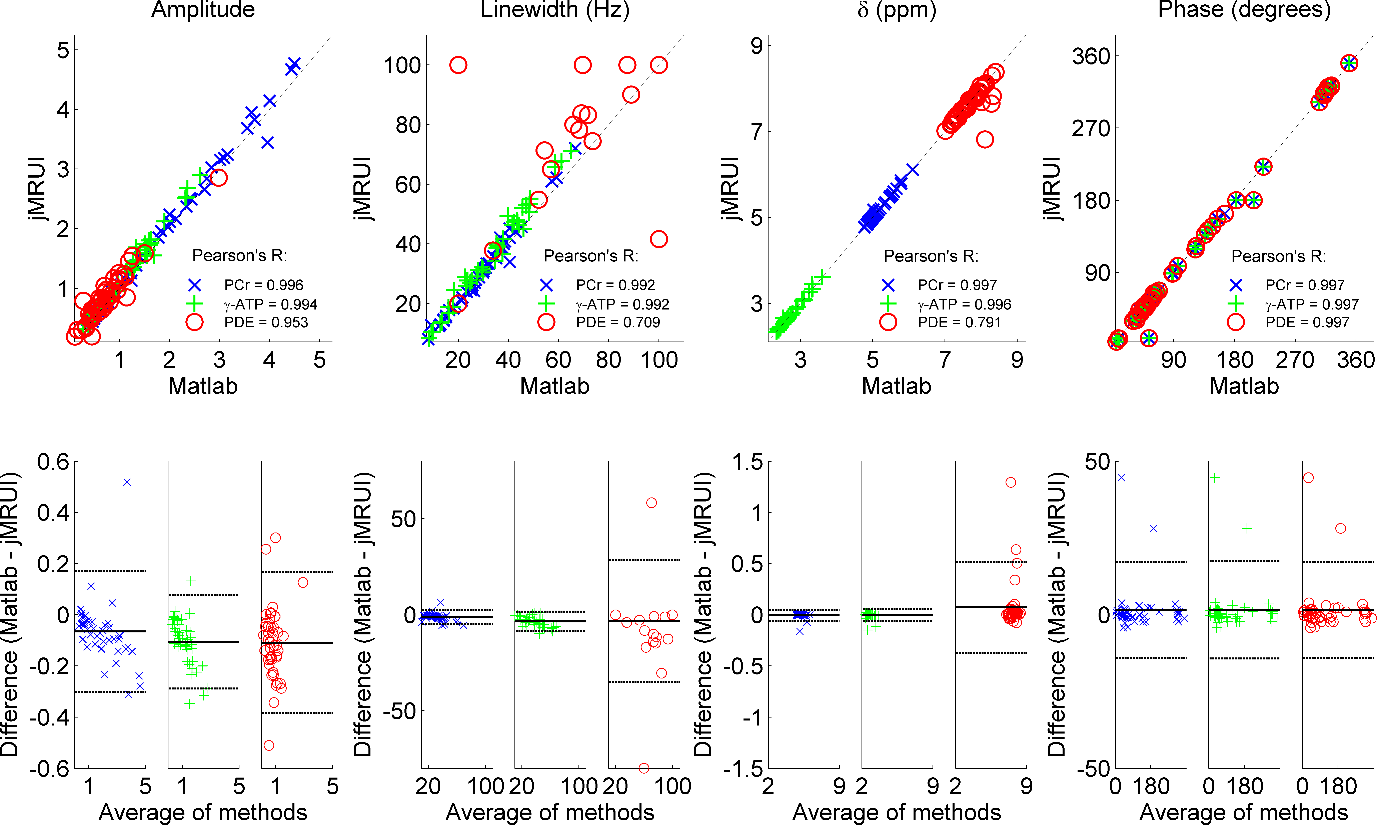


**S4 Fig. Comparison of in vivo fitting by jMRUI and OXSA.** Correlation and Bland-Altman plots for each peak parameter fitted across high (PCr), medium (γ-ATP) and low (PDE) SNR peaks by jMRUI and Matlab (OXSA) for data from the mid-interventricular septal voxel of 45 cardiac ^31^P-MRS datasets, acquired at 3T and 7T. The dashed lines are lines of identity.

Strong correlation (R > 0.95) was also observed between jMRUI and OXSA estimates of the CRLB peak parameters of the PCR and γ-ATP peaks. PDE showed correlations >0.7. All CRLB except the PDE phase and linewidth were significantly smaller (t-test, α = 0.05).

## Discussion

The OXSA results matched jMRUI AMARES well in simulation with less than one percent error at noise variances less than 10^3^. There was also excellent correlation (i.e. R > 0.9) for the in vivo cardiac data, except for the linewidths and chemical shift of PDE (R > 0.7), which is a very low SNR peak. The biggest difference between the two fitting methods was speed. Even though Matlab is an interpreted language and FORTRAN is compiled (and therefore usually faster), our OXSA AMARES implementation benefits from multi-threaded execution in the LAPACK libraries. This accelerates the critical steps in the non-linear least squares fitting and gives quicker fitting than the FORTRAN code in jMRUI. This difference was less obvious for simple spectra (7.77-12.1% reduction in time for doublet and singlet spectra), but became clear for the simulated and in vivo cardiac spectra (93.7-96.3% reduction in time).

## References

1. De Graaf RA. In vivo NMR spectroscopy: principles and techniques. 2nd ed. Chichester: John Wiley & Sons; 2007.

2. Rodgers CT, Clarke WT, Snyder C, Vaughan JT, Neubauer S, Robson MD. Human cardiac 31P magnetic resonance spectroscopy at 7 tesla. Magn Reson Med. 2014; 72(2):304-15.

3. Cavassila S, Deval S, Huegen C, van Ormondt D, Graveron-Demilly D. Cramér-Rao bounds: an evaluation tool for quantitation. NMR Biomed. 2001; 14(4):278-83.

4. Cavassila S, Deval S, Huegen C, van Ormondt D, Graveron-Demilly D. Cramér-Rao bound expressions for parametric estimation of overlapping peaks: Influence of prior knowledge. J Magn Reson. 2000; 143(2):311-20.
